# A dataset for assessing temporal changes in gene expression during the aging process of adult *Drosophila melanogaster*

**DOI:** 10.1016/j.dib.2016.04.072

**Published:** 2016-05-06

**Authors:** Kimberly A. Carlson, Chi Zhang, Lawrence G. Harshman

**Affiliations:** a Biology Department, University of Nebraska at Kearney, Kearney, NE 68849 USA; b School of Biological Sciences, University of Nebraska at Lincoln, Lincoln, NE 68588 USA

**Keywords:** Aging, *Drosophila melanogaster*, Transcriptome, Gene expression, Longitudinal study

## Abstract

A *Drosophila melanogaster* genome-wide transcriptome dataset is available for studies on temporal patterns of gene expression. Gene expression was measured using two-dye color oligonucleotide arrays derived from Version 2 of the *Drosophila* Genomics Resource Center. A total of 15,158 oligonucleotide probes corresponded to a high proportion of the coding genes in the genome. The source of the flies was a highly genetically heterogeneous population maintained in an overlapping generation population regime. This regime was designed to maintain life history traits so that they were similar to those found in natural populations. Flies collected for the cohorts were obtained in a short period of time in a carefully controlled manner before virgin females and males were allowed to mate. Mated females were introduced into two large population cages in unusually high numbers (approximately 12,000 per cage) for a *Drosophila* laboratory longevity study. Samples were taken weekly from each cohort for 11 weeks; only a small proportion of surviving flies were present at the last two collection time points and thus they were exceptionally old compared to those collected in early-to-midlife samples. The data set is useful for studies of temporal patterns of gene expression as flies age. The very large size of each cohort, and relatively frequent incidence of temporal samples, allows for a fine-scale study of gene expression from young to very old flies.

**Specifications Table**

**Table:** 

Subject area	Biology
More specific subject area	cDNA microarray transcriptome analysis
Type of data	Two-color Version 2 DGRC (*Drosophila* Genomics Resource Center) oligonucleotide microarray
How data was acquired	RNA extraction, Bioanalysis of RNA using Agilent 2100 Bioanalyzer, cDNA microarray analysis, data analysis with Linear Models for Microarray Analysis(LIMMA) package in Bioconductor, Gene Set Enrichment Analysis (GSEA)
Data format	Raw data: TAR; Normalized data: SOFT, MINIML, and TXT
Experimental factors	Age and survival
Experimental features	Two 3′x2′x1′ cages with ~12,000 once mated females were sampled weekly for ~11 weeks and cDNA microarray analysis performed on all collections.
Data source location	Kearney, Nebraska, USA
Data accessibility	Data is deposited at http://www.ncbi.nlm.nih.gov/geo/query/acc.cgi?acc=GSE67547

**Value of the data**

• A longitudinal cohort genome-wide transcriptome data set of adult female *D. melanogaster* aging has been generated, which is valuable for future studies of temporal variation of gene expression on a fine temporal scale.
• The source fly population was initiated and maintained in a manner designed to preserve genetic variation relevant to natural populations.
• Highly comparable replicate cohorts were used for the study, each cohort was very large which enabled collection of an extensive series of transcriptomic data points that included very old flies.

## 1 Data

The source of this dataset is a longitudinal study of gene expression in two large laboratory cohorts (http://dx.doi.org/10.1155/2015/835624 [1]). This data originated from cDNA microarray analysis of whole-body RNA samples comparing gene-expression profiles over the lifespan of females from the two large *Drosophila melanogaster* cohorts. Samples of flies were taken from the cohorts as a function of age a relatively large number of times and thus the transcriptome was represented frequently in young to very old flies.

## 2 Experimental design, materials and methods

The design of the published dataset [1] was four-fold. One design component was the use of flies for the transcriptomic longitudinal aging cohort study that were representative of natural genetic variation to the extent possible in a stable laboratory population. A second design feature was to collect uniformly-treated female *D. melanogaster* that were intended to be highly comparable between ages as samples for microarray analysis. A third design objective was to conduct the experiment in replicate cohorts to assess the repeatability of gene expression. A fourth design feature was to initiate two very large cohorts of females to provide a sufficient number of flies for the destructive sampling for each of the weekly collections as flies age. Thus, there were a sufficient number of flies to allow sampling at very old ages. The purpose of this presentation is to emphasize the value of the data set for future studies of temporal patterns of gene expression. For example, in [1] we observed increasing variance of gene expression of a high proportion of 316 immune function genes relatively to genes randomly selected from the genome. This is a unique genome-scale observation of increasing variance of age-dependent gene expression which could represent loss of control of transcription. However, we did not exhaustively investigate other genes with high variance of gene expression as flies aged in the large cohorts and this research could be undertaken in the future. As another example, additional research could be conducted on the oldest female flies sampled in [1], as they are a valuable byproduct of the very large cohorts used in the study. In general, there are a range of opportunities to use the dataset presented in [1], and described here, for future research on temporal patterns of gene expression as *D. melanogaster* age.

### 2.1 Establishment and culture of a source population of flies

The flies for the present study were based on a set of lines derived from a natural population in the University of California Davis Wolfskill Experimental Orchards near Winters, California. Flies collected in the field were inbred by sib mating for 20 generations starting immediately after collection. Twenty inbred lines were crossed in all possible combinations, including reciprocal crosses, and a standard number of progeny per cross used to establish a large laboratory base population of at least 10,000 adults. All possible combinations of crosses refer to a specific all-inclusive regime of mating between the lines. For example, inbred line 1 was reciprocally crossed to lines 2 through 20. Similarly, inbred line 2 was reciprocally crossed to lines 3 through 20. From each cross, 100 progeny were released into a random mixture of flies, which was used to initiate the base population. The number of lines (20) used as a source of genetic variation in this study, and the crossing scheme, was based on the goal of generating a large genetically heterogeneous laboratory base population. This population was maintained in an overlapping generation regime designed to maintain natural genetic variation that supports natural levels of life span and stress resistance both of which otherwise diminish during conventional laboratory culture [2], [3]. The base population was kept in the laboratory for approximately 16 months whereupon it provided the large number of genetically heterogeneous outbred flies used for the two cohorts in the present study. The issue of maintaining natural genetic variation in an equilibrium laboratory population has been considered in relation to selection experiments [4], [5]. The perspectives presented in [4], [5] were the basis for the design of the process of establishment and maintenance of the base population used in this study. The motivation was an attempt to represent natural genetic variation, to the extent possible, in a large long-term (quasi-equilibrium) laboratory population.

### 2.2 Collection of mated females and maintenance in two large cohorts

The flies used to initiate the cohorts for the longitudinal cohort study were generated in a controlled manner from the base population that was being maintained in a 3′x2′x1′ cage. Pint bottles with food were placed in the cages in random locations for one day to collect eggs. From the eggs in these bottles, 100 vials were seeded with 100 eggs/vial. This was repeated the next day to give a total of 200 seeded vials. Vials were maintained at 25 °C with diurnal light until eclosion. All flies obtained from vials were randomized by combining flies into a large bottle and mixing them. This mass assortment of flies was subdivided into a series of bottles. From these bottles, 250 females were placed into each of 10 bottles and allowed to lay eggs. After one day, the females were transferred into a holding bottle and 100 vials were seeded with 100 eggs/vial. After all the eggs were collected, the females were returned to the original bottles. After another day (day 2), the females were once again transferred into a holding bottle and 50 vials were seeded with 100 eggs/vial. After all the eggs were collected, the females were returned to the original bottles. After yet one more day (day 3), the females were once again transferred into a holding bottle and 75 vials were seeded with 100 eggs/vial. After all the eggs were collected, the females were returned to the original bottles. After yet one more day (day 4), the females were once again transferred into a holding bottle and 75 vials were seeded with 100 eggs/vial. After all the eggs were collected, the females were returned to the original bottles. All of the vials with eggs were sent overnight to the University of Nebraska at Kearney. Upon receipt, the seeded vials were placed at 25 °C with diurnal light until eclosion. After eclosion, the flies were lightly etherized, sexed, and sets of 25 of each sex were placed in 8 oz bottles containing food and allowed to mate. A total of 85 bottles were prepared, and the flies in each bottle allowed to lay eggs for 48 h. These flies were twice transferred to bottles with fresh food for the purpose of egg accumulation. The mating and egg-producing sets were held in a laboratory environment at ~22–24 °C with a diurnal light cycle.

The bottles were watched carefully once pupation was evident, and soon after the time of eclosion emergent flies were lightly etherized, sorted by sex, and counted. During the process of eclosion, 75 females or 75 males were placed into individual 8 oz bottles with food, until approximately 25,000 flies were collected (12,500 males and 12,500 females). The females were allowed to mature for 3 days and the males allowed to mature for a minimum of 2 days. After this time period, sets of 75 females and males were allowed to mate for 24 h. After one day of mating, a very high proportion of the females would be at least singly mated and some would have mated more than one time. After mating, the flies were gently etherized, sexed, counted, and males discarded. Approximately 12,000 mated females were released into each of two 3′×2′×1′ Plexiglas cages. Each cage had two holes on either side covered with tubigrip (ConvaTec, Princeton, NJ) to allow access into a cage without the loss of flies. The cages each contained six large (150 x 15  mm) Petri dishes of media and an additional two large Petri dishes containing cotton balls wetted with Nanopure water. There was enough fresh water in each petri plate to provide for easily accessible drinking water and a source of water for humidity. The cages were held in a laboratory at ~22–24 °C with a diurnal light cycle. The media Petri dishes were changed every day, the water checked every day, and water replaced every other day. The cages had their positions changed each day with respect to top or bottom position as they were stacked on top of each other. The purpose of changing the top and bottom position of each cage was to ensure that there was no effect associated with differences in light incidence, or other factors, that varied with respect to top or bottom cage location.

### 2.3 Sample collection and mortality tabulation

The large number of mated female flies released into each population cage allowed for very old flies to be sampled. Sampling for the microarrays was destructive in that the flies had to be frozen as a source of RNA for the microarrays. The last sample was taken 11 weeks after the control flies were collected in each large cage cohort for a total of 12 samples. The initial number of individuals in a cohort for a longitudinal study has been likened to the size of a deep space telescope in the sense that the size of the telescope allows one to look deeper into space and similarly the size of the initial population in a cohort allowed one to investigate older ages in the context of the study design [1]. The number of surviving individuals in the longitudinal study that was sufficient for destructive sampling at the oldest ages was a function of initial number in the cohort.

Each day in each large population cage the dead flies were collected by aspiration and tallied. Mortality curves comparing the number of total dead flies over time were constructed. Transcriptome control time point sexually mature female flies were collected at six days old; flies were four days old before being released into the boxes and after two days residency in the large cages they were sampled for control RNA for the entire study. Flies from this time point were used for the standard sample in the two-sample microarrays used in the present study. In addition to the control samples, twenty-two samples of 24 females each were collected by aspiration, lightly etherized, counted, and allowed to recover for two hours in vials containing fly food. After two hours, the females were flash-frozen in liquid nitrogen, transferred to dry ice, and stored at −80 °C. Every seven days after the collection of the control females, four samples of 12 females each were collected following the same protocol as the control females. The flies were collected at 1:00 pm CST and frozen at 3:00 pm CST. Collection lasted until day 79 when there were only enough females for this last collection. Samples were collected at days 2 (control flies), 9, 16, 23, 30, 37, 44, 51, 58, 65, 72, and 79 in the cages.

### 2.4 RNA extraction, and cDNA microarray hybridization

Total RNA was extracted from all samples utilizing the standard TRIzol protocol (Invitrogen, Carlsbad, CA), cleaned using the Qiagen RNeasy Mini Kit (Qiagen, Valencia, CA), and the quality and integrity of the RNA was assessed at the University of Nebraska Lincoln Genomics Core Facility using an Agilent 2100 Bioanalyzer (Agilent Technologies, Inc., Palo Alto, CA). The University of Nebraska Medical Center (UNMC) Microarray Core Facility used the quantified RNA to perform two-color Version 2 DGRC oligonucleotide microarrays (*Drosophila* Genomics Resource Center [DGRC], Bloomington, IN) consisting of 15, 158 oligonucleotides (~93% of the annotated genes of *D. melanogaster*).

Twelve micrograms of total RNA per sample was indirectly labeled with the Cy3/Cy5 fluorescent dye using the Superscript Indirect cDNA Labeling System for DNA Microarrays (Invitrogen) per manufacturer׳s instructions. This system uses an aminoallyl-modified nucleotide and an aminohexyl-modified nucleotide together with other dNTPs in a cDNA synthesis reaction with SuperScript™ III Reverse Transcriptase (RT). Following reverse transcription, amino-allyl labeled cDNA was incubated with Cy3/Cy5 in DMSO to couple the dyes to the cDNA to create fluorescently labeled probes. These were purified by gel-exclusion chromatography using SNAP columns (Invitrogen). All probes were assayed by spectrophotometry to assess the robustness of the reverse transcription and recovery of the probes, as well as the integrity of the coupling reactions. Only probes that were of sufficient cDNA concentration and specific activity were committed to hybridization to slides. The probes were mixed together in 40 ml hybridization buffer with blocking agents that included poly-dA (20 mg) and *Cot*-*1* DNA (20 mg) added. Microarray slides were pre-hybridized for 45 min at 42 °C in 3x SSC solution plus 1% bovine serum albumin, with hybridization performed overnight at 42 °C. After hybridization, the slides were washed 2x times with 2.0x SSC, 0.5% SDS at 42 °C for 15 min, followed by washing 2x with 0.5x SSC, 0.50% SDS for 15 min each. Cy3 (532 nm) and Cy5 (635 nm) scans were performed using an ScanPix 4000B slide reader per manufacturer׳s suggested conditions (Molecular Devices, Sunnyvale, CA).

### 2.5 cDNA microarray analysis, gene set enrichment analysis (GSEA), and variance in immune function analysis

The initial cDNA microarray analysis consisted of pair-wise comparisons of each time point to the control (after two days in the cage, day six post-eclosion). The control samples of females collected at two days in the cage were used as a common reference for ensuing time-point hybridizations. The later-age samples (post-control) from the two cages were collected at 11 additional time points: 9, 16, 23, 30, 37, 44, 51, 58, 65, 72, and 79 days in the cages. Analyses were conducted with Linear Models for Microarray Analysis (LIMMA) package in Bioconductor [6], [7], [8]. The genes identified as differentially expressed across all the time points were subjected to cluster analyses. The GeneCluster 2 package, a self-organizing map (SOM) clustering algorithm [9], was applied to the significantly differentially expressed genes. From this, self-organizing map (SOM) and hierarchical clustering heatmaps (correlation-based distance, average link) were generated. The identified differentially expressed genes in each cluster were subjected to ontology analyses using PANTHER (Protein ANalysis THrough Evolutionary Relationships; http://www.pantherdb.org [10], [11], as previously described [1].

GSEA (Gene Set Enrichment Analysis) was performed as previously described [1], employing Bioconductor packages [6], [12] for quality assurance, and background correction, normalization, empirical Bayes correction, and the calculation of statistical significance for differential gene expression was performed by using the LIMMA package [8]. For multiple test correction, Benjamini and Hochberg׳s False Discovery Rate was used [13]. The KEGG (Kyoto Encyclopedia of Genes and Genomes) Database of Biochemical Pathways [14] and the Gene Ontology (GO) categories for biological processes, molecular functions and cellular localizations [15] were used and the statistical significance of enrichment of a gene set in either the up-regulated or down-regulated genes were calculated using GSEA [16]. Transcript level patterns across the seventy-nine day time span of the experiments relative to the control samples were assessed by *k*-means cluster analysis using different numbers of clusters. The consistency of transcript level changes through time was evaluated by the MATLAB implementation of the biclustering method which is clustering the base 2 logarithm fold change values [12]. Biclustering, with minor exceptions, faithfully reproduced time points in chronological order.

The expression variance of a gene was calculated as the standard deviation of average gene expression derived from the three replicates that were taken for each time point. Based on the relatively high incidence of immunity genes in clusters of differentially expressed genes, 316 immune function genes were selected for the analysis of an extended set of functionally related genes that could have exhibited increasing variance in gene expression as a function of age. Variance in expression of immune genes was calculated within cages (Boxes A and B), and among cages, which was based on a mixture of samples from Box A and Box B. For a comparison, 200 genes, excluding immune function genes, were randomly selected from the entire *D. melanogaster* genome, and the variance in expression of those genes was calculated among cages. A linear model was fitted between the variance of expression and time points to find their trend.
